# Deep Neural Networks Based Recognition of Plant Diseases by Leaf Image Classification

**DOI:** 10.1155/2016/3289801

**Published:** 2016-06-22

**Authors:** Srdjan Sladojevic, Marko Arsenovic, Andras Anderla, Dubravko Culibrk, Darko Stefanovic

**Affiliations:** ^1^Department of Industrial Engineering and Management, Faculty of Technical Sciences, University of Novi Sad, Trg Dositeja Obradovica 6, 21000 Novi Sad, Serbia; ^2^Department of Information Engineering and Computer Science, University of Trento, Via Sommarive 9, Povo, 38123 Trento, Italy

## Abstract

The latest generation of convolutional neural networks (CNNs) has achieved impressive results in the field of image classification. This paper is concerned with a new approach to the development of plant disease recognition model, based on leaf image classification, by the use of deep convolutional networks. Novel way of training and the methodology used facilitate a quick and easy system implementation in practice. The developed model is able to recognize 13 different types of plant diseases out of healthy leaves, with the ability to distinguish plant leaves from their surroundings. According to our knowledge, this method for plant disease recognition has been proposed for the first time. All essential steps required for implementing this disease recognition model are fully described throughout the paper, starting from gathering images in order to create a database, assessed by agricultural experts. Caffe, a deep learning framework developed by Berkley Vision and Learning Centre, was used to perform the deep CNN training. The experimental results on the developed model achieved precision between 91% and 98%, for separate class tests, on average 96.3%.

## 1. Introduction

The problem of efficient plant disease protection is closely related to the problems of sustainable agriculture and climate change [1]. Research results indicate that climate change can alter stages and rates of pathogen development; it can also modify host resistance, which leads to physiological changes of host-pathogen interactions [2, 3]. The situation is further complicated by the fact that, today, diseases are transferred globally more easily than ever before. New diseases can occur in places where they were previously unidentified and, inherently, where there is no local expertise to combat them [4–6].

Inexperienced pesticide usage can cause the development of long-term resistance of the pathogens, severely reducing the ability to fight back. Timely and accurate diagnosis of plant diseases is one of the pillars of precision agriculture [7]. It is crucial to prevent unnecessary waste of financial and other resources, thus achieving healthier production, by addressing the long-term pathogen resistance development problem and mitigating the negative effects of climate change.

In this changing environment, appropriate and timely disease identification including early prevention has never been more important. There are several ways to detect plant pathologies. Some diseases do not have any visible symptoms, or the effect becomes noticeable too late to act, and in those situations, a sophisticated analysis is obligatory. However, most diseases generate some kind of manifestation in the visible spectrum, so the naked eye examination of a trained professional is the prime technique adopted in practice for plant disease detection. In order to achieve accurate plant disease diagnostics a plant pathologist should possess good observation skills so that one can identify characteristic symptoms [8]. Variations in symptoms indicated by diseased plants may lead to an improper diagnosis since amateur gardeners and hobbyists could have more difficulties determining it than a professional plant pathologist. An automated system designed to help identify plant diseases by the plant's appearance and visual symptoms could be of great help to amateurs in the gardening process and also trained professionals as a verification system in disease diagnostics.

Advances in computer vision present an opportunity to expand and enhance the practice of precise plant protection and extend the market of computer vision applications in the field of precision agriculture.

Exploiting common digital image processing techniques such as colour analysis and thresholding [9] were used with the aim of detection and classification of plant diseases.

Various different approaches are currently used for detecting plant diseases and most common are artificial neural networks (ANNs) [10] and Support Vector Machines (SVMs) [11]. They are combined with different methods of image preprocessing in favour of better feature extraction.

In machine learning and cognitive science, ANN is an information-processing paradigm that was inspired by the way biological nervous systems, such as the brain, process information. The brain is composed of a large number of highly interconnected neurons working together to solve specific problems.

An artificial neuron is a processing element with many inputs and one output. Although artificial neurons can have many outputs, only those with exactly one output will be considered. Their inputs can also take on any value between 0 and 1. Also, the neuron has weights for each input and an overall bias.

The weights are real numbers expressing importance of the respective inputs to the output. The bias is used for controlling how easy the neuron is getting to output 1. For a neuron with really big bias it is easy to output 1, but when the bias is very negative then it is difficult to output 1.

The output of the neuron is not 0 or 1. Instead, it is *α* · (*w* · *x* + *b*), where *α* is called the transfer function. There are different types of transfer function: step, linear, sigmoid, and so forth. The smoothness of *α* means that small changes Δ*w*_*j*_ in the weights and Δ*b* in the bias will produce small change Δoutput in the output from the neuron. Small output change is approximated by

(1) $$\begin{array}{c} \Delta output\approx \sum \frac{\partial \text{output}}{\partial w_{j}}{\Delta w}_{j}+\sum \frac{\partial \text{output}}{\partial b}\Delta b. \end{array}$$

**Figure 1:**
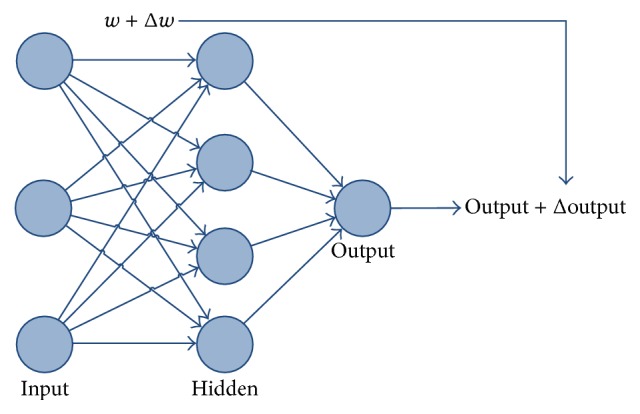
Simple model of ANN.

Basically, the small change in weight or bias causes the small corresponding change in the network output (Figure 1).

Neural networks, with their outstanding ability to derive meaning from complex or imperfect data, can be applied for extracting patterns and detecting trends that are too difficult to notice by humans or computer techniques. Other advantages of ANNs are adaptive learning, self-organization, real time operations, and so forth.

There are two main categories of ANNs when speaking about architecture: feed-forward ANNs where the output of any layer is unlikely to influence that same layer and feedback ANNs where signals travel in both directions by involving loops in the network.

The method described in this paper is a new approach in detecting plant diseases using the deep convolutional neural network trained and fine-tuned to fit accurately to the database of a plant's leaves that was gathered independently for diverse plant diseases. The advance and novelty of the developed model lie in its simplicity; healthy leaves and background images are in line with other classes, enabling the model to distinguish between diseased leaves and healthy ones or from the environment by using deep CNN.

The rest of the paper is organized as follows: Section 2 presents related work, Section 3 presents methodology, Section 4 presents achieved results and related discussion, and finally, Section 5 holds our conclusions.

## 2. Related Work

Implementing the appropriate management strategies like fungicide applications, disease-specific chemical applications, and vector control through pesticide applications could lead to early information on crop health and disease detection. This could facilitate the control of diseases and improve productivity. In [12], authors present, review, and recognize the demand for developing a rapid, cost-effective, and reliable health-monitoring sensor that facilitates advancements in agriculture. They described the currently used technologies that include spectroscopic and imaging-based and volatile profiling-based plant disease detection methods for the purpose of developing ground-based sensor system to assist in monitoring health and diseases in plants under field conditions.

After analysis of their work and analysis presented by the authors of [13–16], it was decided to use image processing disease recognition approach among other approaches commonly used for plant disease diagnostics, for instance, double-stranded ribonucleic acid (RNA) analysis, nucleic acid probes, and microscopy.

Numerous procedures are currently in use for plant disease detection applying computer vision. One of them is disease detection by extracting colour feature as authors in [17] have presented. In this paper YcbCr, HSI, and CIELB colour models were used in the study; as a result, disease spots were successfully detected and remained unaffected by the noise from different sources, such as camera flash.

In addition, plant disease detection could be achieved by extracting shape features method. Patil and Bodhe applied this technique for disease detection in sugarcane leaves where they have used threshold segmentation to determine leaf area and triangle threshold for lesioning area, getting the average accuracy of 98.60% at the final experiments [18].

Furthermore, extracting texture feature could be used in detecting plant diseases. Patil and Kumar proposed a model for plant disease detection using texture features such as inertia, homogeneity, and correlation obtained by calculating the gray level cooccurrence matrix on image [19]. Combined with colour extraction, they experimented on detecting diseases on maize leaves.

Combination of all these features provides a robust feature set for image improvement and better classification. In [20], the authors have presented a survey of well-known conventional methods of feature extraction. Due to the rapid progress of Artificial Intelligence (AI) science, work in this paper is mainly focused on applying these methodologies and techniques.

There are some approaches which apply the feed-forward back propagation of neural networks consisting of one input, one output, and one hidden layer for the needs of identifying the species of leaf, pest, or disease; this model was proposed by the authors in [21]. They developed a software model, to suggest remedial measures for pest or disease management in agricultural crops.

Another technique proposed by the authors in [22] incorporates the features extracted by Particle Swarm Optimization (PSO) [23] and forward neural network in direction of determining the injured leaf spot of cotton and improving the accuracy of the system with the final overall accuracy of 95%.

Also, detection and differentiation of plant diseases can be achieved using Support Vector Machine algorithms. This technique was implemented for sugar beet diseases and presented in [24], where, depending on the type and stage of disease, the classification accuracy was between 65% and 90%.

Likewise, there are methods that combine the feature extraction and Neural Network Ensemble (NNE) for plant disease recognition. Through training a definite number of neural networks and combining their results after that, NNE offers a better generalization of learning ability [25]. Such method was implemented only for recognizing tea leaf diseases with final testing accuracy of 91% [26].

Another approach based on leaf images and using ANNs as a technique for an automatic detection and classification of plant diseases was used in conjunction with *K*-means as a clustering procedure proposed by the authors in [27]. ANN consisted of 10 hidden layers. The number of outputs was 6 which was the number of classes representing five diseases along with the case of a healthy leaf. On average, the accuracy of classification using this approach was 94.67%.

The authors in [28–31] presented the deep learning methods for solving most complex tasks in different areas of research in biology, bioinformatics, biomedicine, robotics, and 3D technologies.

In our study, we exploit the deep learning method for plant disease recognition, driven by evolvement of deep learning techniques and their application in practice. Extensive search of the state-of-the-art literature yielded no evidence that researchers explored deep learning approach for plant diseases recognition from the leaf images. Our method of recognition by applying deep CNN is presented in the sections below.

## 3. Materials and Methods

The entire procedure of developing the model for plant disease recognition using deep CNN is described further in detail. The complete process is divided into several necessary stages in subsections below, starting with gathering images for classification process using deep neural networks.

### 3.1. Dataset

Appropriate datasets are required at all stages of object recognition research, starting from training phase to evaluating the performance of recognition algorithms. All the images collected for the dataset were downloaded from the Internet, searched by disease and plant name on various sources in different languages, such as Latin, English, German, Serbian, and Hungarian. Images in the dataset were grouped into fifteen different classes. Thirteen classes represented plant diseases which could be visually determined from leaves.

In order to distinguish healthy leaves from diseased ones, one more class was added in the dataset. It contains only images of healthy leaves. An extra class in the dataset with background images was beneficial to get more accurate classification. Thus, deep neural network could be trained to differentiate the leaves from the surrounding. The background images were taken from the Stanford background dataset [32].

In this stage, all duplicated images taken from different sources were removed by developed python script applying the comparing procedure. The script removed the duplicates by comparing the images' metadata: name, size, and the date. After the automated removal, images were assessed by human experts in much iteration.

Next step was to enrich the dataset with augmented images. The main goal of the presented study is to train the network to learn the features that distinguish one class from the others. Therefore, when using more augmented images, the chance for the network to learn the appropriate features has been increased. Finally, a database containing 30880 images for training and 2589 images for validation has been created. The augmentation process is described in Section 3.3.

Table 1 shows all supported diseases together with the number of original images and number of augmented images for every class used as training and validation dataset for the disease classification model.

### 3.2. Image Preprocessing and Labelling

Images downloaded from the Internet were in various formats along with different resolutions and quality. In order to get better feature extraction, final images intended to be used as dataset for deep neural network classifier were preprocessed in order to gain consistency. Furthermore, procedure of image preprocessing involved cropping of all the images manually, making the square around the leaves, in order to highlight the region of interest (plant leaves). During the phase of collecting the images for the dataset, images with smaller resolution and dimension less than 500 px were not considered as valid images for the dataset. In addition, only the images where the region of interest was in higher resolution were marked as eligible candidates for the dataset. In that way, it was ensured that images contain all the needed information for feature learning. Images used for the dataset were image resized to 256 × 256 to reduce the time of training, which was automatically computed by written script in Python, using the OpenCV framework [33].

Many resources can be found by searching across the Internet, but their relevance is often unreliable. In the interest of confirming the accuracy of classes in the dataset, initially grouped by a keywords search, agricultural experts examined leaf images and labelled all the images with appropriate disease acronym. As it is known, it is important to use accurately classified images for the training and validation dataset. Only in that way may an appropriate and reliable detecting model be developed. In this stage, duplicated images that were left after the initial iteration of gathering and grouping images into classes described in Section 3.1 were removed from the dataset.

### 3.3. Augmentation Process

The main purpose of applying augmentation is to increase the dataset and introduce slight distortion to the images which helps in reducing overfitting during the training stage. In machine learning, as well as in statistics, overfitting appears when a statistical model describes random noise or error rather than underlying relationship [34]. The image augmentation contained one of several transformation techniques including affine transformation, perspective transformation, and simple image rotations. Affine transformations were applied to express translations and rotations (linear transformations and vector addition, resp.) [35] where all parallel lines in the original image are still parallel in the output image. To find a transformation matrix, three points from the original image were needed as well as their corresponding locations in the output image. For perspective transformation, a 3 × 3 transformation matrix was required. Straight lines would remain straight even after the transformation. For the augmentation process, simple image rotations were applied, as well as rotations on the different axis by various degrees.

**Figure 2:**
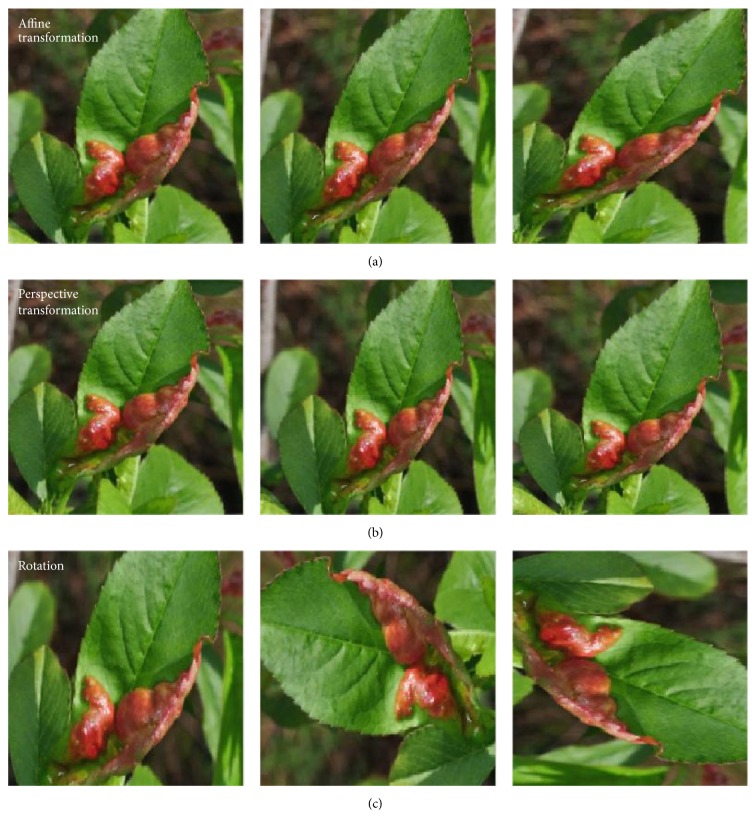
Image transformations used for augmentation: (a) affine transformations; (b) perspective transformations; (c) rotations.

Transformations applied in augmentation process are illustrated in Figure 2, where the first row represents resulting images obtained by applying affine transformation on the single image; the second row represents images obtained from perspective transformation against the input image and the last row visualizes the simple rotation of the input image. The process of augmentation was chosen to fit the needs; the leaves in a natural environment could vary in visual perspective.

For this stage, in order to automate the augmentation process for numerous images from the dataset, particular application was developed in C++ using the OpenCV library [36], with possibility of changing the parameters of transformation during the run-time, which improves flexibility.

### 3.4. Neural Network Training

Training the deep convolutional neural network for making an image classification model from a dataset described in Section 3.1 was proposed. There are several well-known state-of-the-art deep learning frameworks, such as Python library Theano [37] and machine learning library that extends Lua, Torch7 [38]. In addition, there is Caffe, an open source deep learning framework developed by the BVLC [39] containing reference pretrained CaffeNet model. For the purpose of this research, this framework was used, along with the set of weights learned on a very large dataset, ImageNet [40].

Caffe framework is suitable for both research experiments and industry deployment. The core of framework is developed in C++ and provides command line, Python, and MATLAB interfaces. Caffe's integration with cuDNN library accelerates Caffe models [41, 42]. CaffeNet is a deep CNN which has multiple layers that progressively compute features from input images [43]. Specifically, the network contains eight learning layers and five convolutional and three fully connected layers [44].

CaffeNet architecture is considered a starting point, but modified and adjusted to support our 15 categories (classes). Last layer was altered and the output of the softmax layer was parameterized to the requirements of presented study.

The convolutional layer is the essential building block of the convolutional neural network. The layer's parameters are comprised of a set of learnable kernels which possess a small receptive field but extend through the full depth of the input volume [45].

Each convolutional layer has *M* maps of equal size, *M*_*x*_ and *M*_*y*_, and a kernel of size *K*_*x*_, and *K*_*y*_ is shifted over the certain region of the input image. The skipping factors *S*_*x*_ and *S*_*y*_ define how many pixels the filter/kernel skips in *x*- and *y*-direction between subsequent convolutions [46]. The size of the output map could be defined as

(2) Mxn=Mxn−1−KxnSxn+1+1,Myn=Myn−1−KynSyn+1+1,

where *n* indicates the layer. Each map in layer *L* ^*n*^ is connected to most *M* ^*n*−1^ maps in layer *L* ^*n*−1^.

Rectified Linear Units (ReLU) are used as substitute for saturating nonlinearities. This activation function adaptively learns the parameters of rectifiers and improves accuracy at negligible extra computational cost [47]. It is defined as

(3) $$\begin{array}{c} f\left(\;z_{i}\;\right)=\max\left(\;0,z_{i}\;\right), \end{array}$$

where *z*_*i*_ represents the input of the nonlinear activation function *f* on the *i*th channel.

Deep CNN with ReLUs trains several times faster. This method is applied to the output of every convolutional and fully connected layer.

**Figure 3:**
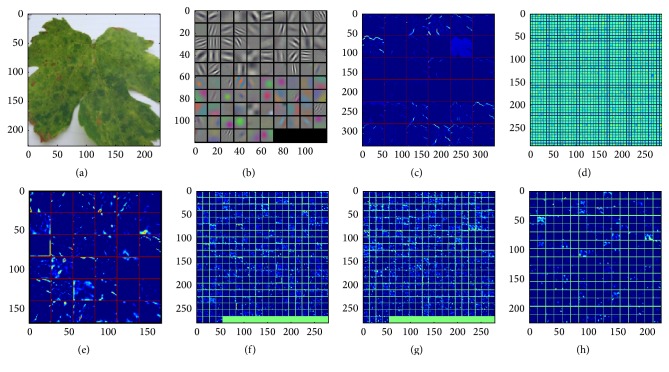
Visualization of features in trained classification model: (a) original image; (b) the first layer filters, Conv1; (c) the first layer output, Conv1 rectified responses of the filters, first 36 only; (d) the second layer filters, Conv2; (e) the second layer output, Conv2 (rectified, only the first 36 of 256 channels); (f) the third layer output, Conv3 (rectified, all 384 channels); (g) the fourth layer output, Conv4 (rectified, all 384 channels); (h) the fifth layer output, Conv5 (rectified, all 256 channels).

Despite the output, the input normalization is not required; it is applied after ReLU nonlinearity after the first and second convolutional layer because it reduces top-1 and top-5 error rates. In CNN, neurons within a hidden layer are segmented into “feature maps.” The neurons within a feature map share the same weight and bias. The neurons within the feature map search for the same feature. These neurons are unique since they are connected to different neurons in the lower layer. So for the first hidden layer, neurons within a feature map will be connected to different regions of the input image. The hidden layer is segmented into feature maps where each neuron in a feature map looks for the same feature but at different positions of the input image. Basically, the feature map is the result of applying convolution across an image. Each layer's features are displayed in a different block, where visualization represents the strongest activation for the provided feature map, starting from the first convolutional layer, where features go from individual pixels to simple lines, to the fifth convolutional layer where learned features like shapes and certain parts of leaves are displayed (Figure 3).

Another important layer of CNNs is the pooling layer, which is a form of nonlinear downsampling. Pooling operation gives the form of translation invariance [48]; it operates independently on every depth slice of the input and resizes it spatially. Overlapping pooling is beneficially applied to lessen overfitting. Also in favour of reducing overfitting, a dropout layer [49] is used in the first two fully connected layers. But the shortcoming of dropout is that it increases training time 2-3 times comparing to a standard neural network of the exact architecture [50]. Bayesian optimization experiments also proved that ReLUs and dropout have synergy effects, which means that it is advantageous when they are used together [51].

The advance of CNNs refer to their ability to learn rich mid-level image representations as opposed to hand-designed low-level features used in other image classification methods [52].

**Figure 4:**
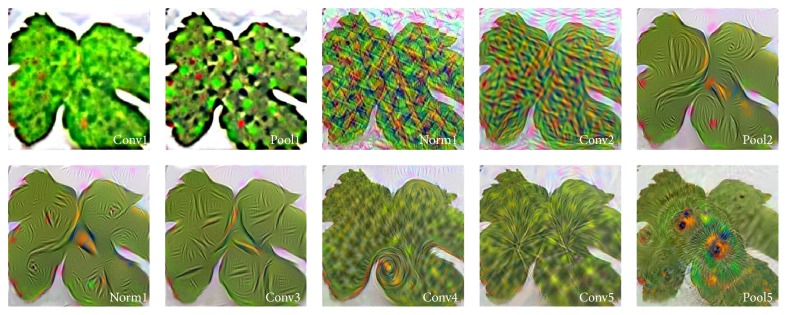
Output layers images.

Figure 4 illustrates the filtered output images after every convolutional and pooling layer of the deep network. Output images are labelled with the name of corresponding layer at the bottom right corner of every image.

### 3.5. Performed Tests

The common approach in measuring performance of artificial neural networks is splitting data into the training set and the test set and then training a neural network on the training set and using the test set for prediction. Thus, since the original outcomes for the testing set and our model predicted outcomes are known, the accuracy of our prediction can be calculated. Different tests were performed with 2589 original images, when trained with 30880 images from database.

For the accuracy test, 10-fold cross validation technique was used to evaluate a predictive model. The cross validation procedure was repeated after every thousand training iteration. Overall estimated result of the test is graphically represented as top-1, to test if the top class (the one having the highest probability) is the same as the target label. The top-5 error rate is there to test if the target label is one of the top 5 predictions, the ones with 5 of the highest probabilities. The number of images used for the validation test from each labelled class is given in Table 1. Test results are presented in Section 4, for both complete dataset and each class separately.

### 3.6. Fine-Tuning

Fine-tuning seeks to increase the effectiveness or efficiency of a process or function by making small modifications to improve or optimize the outcome. The classification function in the original CaffeNet model is softmax classifier that computes probability of 1,000 classes of the ImageNet dataset. Fine-tuned learning experiments require a bit of learning, but they are still much faster than learning from scratch [53]. To start the fine-tuning procedure, this softmax classifier was removed, as mentioned and illustrated in Section 3.4 and the new one was initialized with random values. The new softmax classifier was trained from scratch using the back-propagation algorithm with data from the dataset described in Section 3.1. This dataset has 15 different categories [43]. Due to the smaller size of the dataset used for this research when compared to ImageNet, ILSVRC-2012 [54], overfitting was constrained by using lower initial learning rates for the fine-tuned hidden layers [55]. The learning rate of the top layer was set to 10, while the learning rate of all the other seven learning layers was 0.1. The back-propagation algorithm ran for 100,000 iterations. The process of fine-tuning was repeated changing parameters of hidden layers and hyperparameters. The best suited model for plant disease detection was achieved through the process of experimental adjustment of the parameters. The results of the model fine-tuning are presented and explained further in Section 4.

### 3.7. Equipment

A single PC was used for the entire process of training and testing the plant disease detection model described in this paper. Training of the CNN was performed in Graphics Processing Unit (GPU) mode. Every training iteration took approximately eight hours on this specified machine whose basic characteristics are presented in Table 2.

## 4. Results and Discussion

The results presented in this section are related to training with the whole database containing both original and augmented images. As it is known that convolutional networks are able to learn features when trained on larger datasets, results achieved when trained with only original images will not be explored.

**Figure 5:**
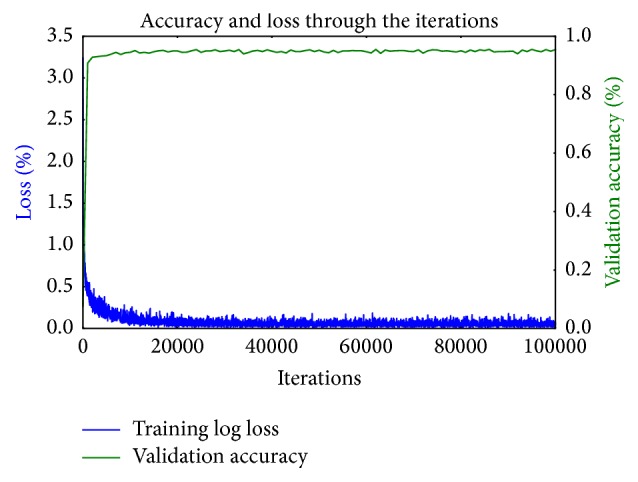
Accuracy of the fine-tuned CNN.

After fine-tuning the parameters of the network, an overall accuracy of 96.3% was achieved, after the 100th training iteration (95.8% without fine-tuning). Even after the 30th training iteration high accuracy results were achieved with exceedingly reduced loss, but after the 60th iteration, the balance in accuracy and loss was carried out in high accuracy. The green line in the graph in Figure 5 shows the network's success on the validation test set, through training iterations. After every 10 thousand training iterations, the snapshot of the model was obtained. The blue line in the graph represents the loss during the training stage. Through training iterations, loss was rapidly reduced.

**Figure 6:**
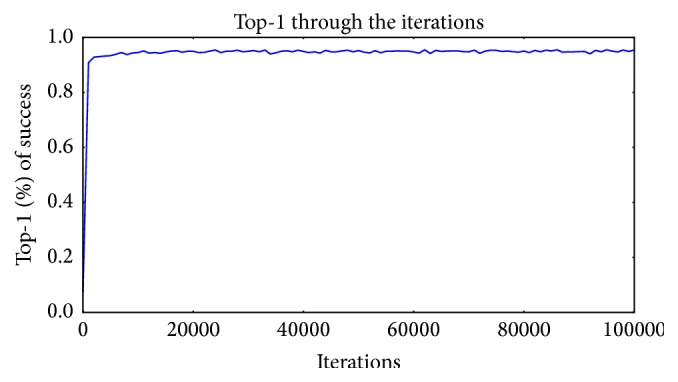
Top-1 accuracy success.

**Figure 7:**
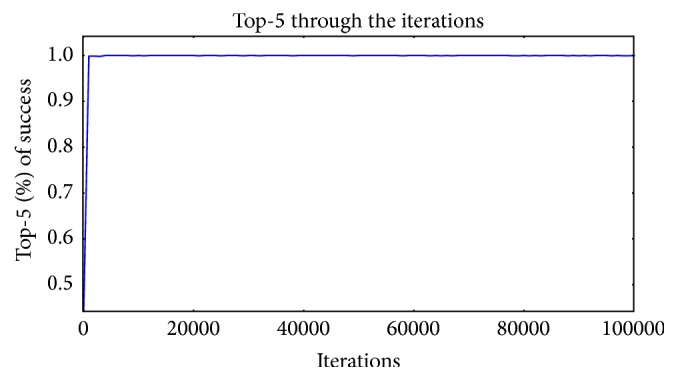
Top-5 accuracy success.

Top-1 success was 96.3% and top-5 success was 99.99% after 100,000 iterations which are shown in Figures 6 and 7, respectively.

**Figure 8:**
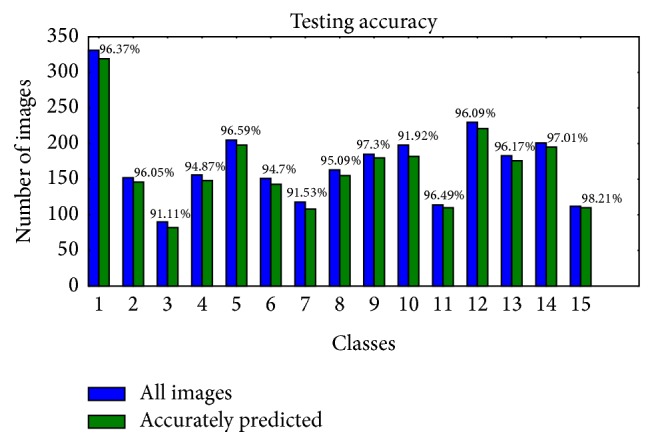
Prediction accuracy for each class separately.

Furthermore, the trained model was tested on each class individually. Test was performed on every image from the validation set. The results are displayed to emphasize how many images from total of each class are accurately predicted.  Figure 8 illustrates trained model's prediction results separated for every class. The class numbers follow enumeration from Table 1.

From the results displayed in Figure 8, it is notable that the trained model's accuracy was slightly less for classes with lower number of images in the training dataset, more specifically classes peach, powdery mildew, apple, powdery mildew, and grapevine, wilt. Achieved accuracy was in range from 91.11% for peach, powdery mildew, up to 98.21% for background images. High accuracy of model's prediction of background images allows good separation of plants leaves and the surroundings.

As suggested by good practice principles, achieved results should be compared with some other results. Taking into account the fact that during this research our own image database was developed, no one has used it up to now. In addition, since no one has used deep learning to identify plant diseases in scientific literature, it is impossible to compare it with other examples. Nonetheless, as a result of extensive review, deep learning techniques have showed better results in pattern recognition, in the image segmentation and object detection. This is also proven in practice by numerous competitions won by convolutional neural networks [56]. Presently, there is a commercial solution, Leafsnap [57], which uses visual recognition in order to identify tree species from their leaves' images but as the network presented in this paper is classifying the plant diseases instead of types of plant, Leafsnap was not used for comparison of the achieved results. Finally, comparing our results with other methods of detecting diseases from leaves images, it can be said that our method provides better results [23, 24, 26, 27].

## 5. Conclusions

There are many methods in automated or computer vision plant disease detection and classification process, but still, this research field is lacking. In addition, there are still no commercial solutions on the market, except those dealing with plant species recognition based on the leaves images.

In this paper, a new approach of using deep learning method was explored in order to automatically classify and detect plant diseases from leaf images. The developed model was able to detect leaf presence and distinguish between healthy leaves and 13 different diseases, which can be visually diagnosed. The complete procedure was described, respectively, from collecting the images used for training and validation to image preprocessing and augmentation and finally the procedure of training the deep CNN and fine-tuning. Different tests were performed in order to check the performance of newly created model.

New plant disease image database was created, containing more than 3,000 original images taken from the available Internet sources and extended to more than 30,000 using appropriate transformations. The experimental results achieved precision between 91% and 98%, for separate class tests. The final overall accuracy of the trained model was 96.3%. Fine-tuning has not shown significant changes in the overall accuracy, but augmentation process had greater influence to achieve respectable results.

As the presented method has not been exploited, as far as we know, in the field of plant disease recognition, there was no comparison with related results, using the exact technique. In comparison with other techniques used and presented in Section 2, comparable or even better results were achieved, especially when taking into account the wider number of classes in the presented study.

An extension of this study will be on gathering images for enriching the database and improving accuracy of the model using different techniques of fine-tuning and augmentation.

The main goal for the future work will be developing a complete system consisting of server side components containing a trained model and an application for smart mobile devices with features such as displaying recognized diseases in fruits, vegetables, and other plants, based on leaf images captured by the mobile phone camera. This application will serve as an aid to farmers (regardless of the level of experience), enabling fast and efficient recognition of plant diseases and facilitating the decision-making process when it comes to the use of chemical pesticides.

Furthermore, future work will involve spreading the usage of the model by training it for plant disease recognition on wider land areas, combining aerial photos of orchards and vineyards captured by drones and convolution neural networks for object detection. By extending this research, the authors hope to achieve a valuable impact on sustainable development, affecting crop quality for future generations.

## Figures and Tables

**Table 1:** Dataset for image classification of leaf disease.

Class	Number of original images	Total number of images: original and augmented	Number of images from the dataset used for validation
(1) Healthy leaf	565	4523	331
(2) Pear, cherry, and peach, porosity	265	2124	152
(3) Peach, powdery mildew	108	1296	90
(4) Peach, *Taphrina deformans*	152	1552	156
(5) Apple, pear, *Erwinia amylovora*	232	2368	205
(6) Apple, pear, *Venturia*	183	2200	151
(7) Apple, powdery mildew	120	1440	118
(8) Apple, Rust	163	1960	163
(9) Pair, *Gymnosporangium sabinae*	267	2142	185
(10) Pair, gray leaf spot	122	1464	198
(11) Grapevine, wilt	287	2300	114
(12) Grapevine, mites	250	2000	230
(13) Grapevine, powdery mildew	237	1900	183
(14) Grapevine, downy mildew	297	2376	201
(15) Background images	1235	1235	112

	**4483**	**30880**	**2589**

**Table 2:** Basic machine characteristics.

Hardware and software	Characteristics
(1) Memory	16 Gb
(2) Processor	Intel Core i7-4790 CPU @ 3.60 GHz ×8
(3) Graphics	GeForce GTX TITAN X 12 Gb
(4) Operating system	Linux Ubuntu 14.04 64 bits
